# A glimpse of the prokaryotic diversity of the Large Aral Sea reveals novel extremophilic bacterial and archaeal groups

**DOI:** 10.1002/mbo3.850

**Published:** 2019-05-06

**Authors:** Vyacheslav Shurigin, Anna Hakobyan, Hovik Panosyan, Dilfuza Egamberdieva, Kakhramon Davranov, Nils‐Kåre Birkeland

**Affiliations:** ^1^ Department of Biological Sciences University of Bergen Bergen Norway; ^2^ Department of Microbiology and Biotechnology, Faculty of Biology National University of Uzbekistan Tashkent Uzbekistan; ^3^ Department of Biochemistry, Microbiology and Biotechnology Yerevan State University Yerevan Armenia; ^4^ Key Laboratory of Biogeography and Bioresource in Arid Land Xinjiang Institute of Ecology and Geography, CAS Urumqi People’s Republic of China; ^5^ Leibniz Centre for Agricultural Landscape Research (ZALF) Müncheberg Germany; ^6^ Present address: Max Planck Institute for Terrestrial Microbiology Marburg Germany

**Keywords:** archaea, bacteria, halophiles, phylogeny, prokaryotic diversity, salt lake

## Abstract

During the last five decades, the Aral Sea has gradually changed from a saline water body to a hypersaline lake. Microbial community inhabiting the Aral Sea has been through a succession and continuous adaptation during the last 50 years of increasing salinization, but so far, the microbial diversity has not been explored. Prokaryotic diversity of the Large Aral Sea using cultivation‐independent methods based on determination of environmental 16S rRNA gene sequences revealed a microbial community related to typical marine or (hyper) saline‐adapted Bacteria and Archaea. The archaeal sequences were phylogenetically affiliated with the order Halobacteriales, with a large number of operational taxonomic units constituting a novel cluster in the Haloferacaceae family. Bacterial community analysis indicated a higher diversity with representatives belonging to Proteobacteria, Actinobacteria and Bacteroidetes. Many members of Alphaproteobacteria and Gammaproteobacteria were affiliated with genera like *Roseovarius, Idiomarina* and *Spiribacter* which have previously been found in marine or hypersaline waters. The majority of the phylotypes was most closely related to uncultivated organisms and shared less than 97% identity with their closest match in GenBank, indicating a unique community structure in the Large Aral Sea with mostly novel species or genera.

## 1 INTRODUCTION

Hypersaline ecosystems such as salt lakes are distributed globally, but differ from each other in terms of salt concentration and chemical composition as determined by local geological characteristics (de la Haba, Sánchez‐Porro, Marquez, & Ventosa, 2011; Naghoni et al., 2017; Simachew, Lanzen, Gessesse, & Øvreas, 2016; Tazi, Breakwell, Harker, & Crandall, 2014). In addition to being hypersaline, these ecosystems are often characterized by other environmental extremes such as high alkalinity, low oxygen concentration and high UV irradiation (Edwardson & Hollibaugh, 2018; Fernandez et al., 2014; Javor, 1989; Naghoni et al., 2017). Hypersaline lakes offer a unique environment for microbial life and are considered as hot spots of microbial diversity (Ley et al., 2006; Oren, 2008). Microbes are fundamental components of hypersaline aquatic ecosystems and play essential roles in global biogeochemical cycles (Sorokin et al., 2014; Yang, Ma, Jiang, Wu, & Dong, 2016).

Most culture‐independent 16S rRNA based studies performed in hypersaline environments worldwide indicate a surprisingly high microbial diversity and abundance of still uncharacterized halophilic microbes (Vavourakis, 2016). Recent investigations of microbial communities from saline lakes such as the Dead Sea, the Great Salt Lake, Middle East soda lakes, African and Antarctic saline lakes have revealed the presence of all taxonomic domains, including Bacteria, Archaea, viruses and eukaryotes (Abdallah et al., 2016; Boutaiba, Hacene, Bidle, & Maupin‐Furlow, 2011; Heidelberg et al., 2013; Lanzen et al., 2013; Oren, Baxter, & Weimer, 2009; Tazi et al., 2014; Ventosa, Fernández, León, Sánchez‐Porro, & Rodriguez‐Valera, 2014). The most abundant bacterial phyla found in hypersaline environments are Proteobacteria, Bacteroidetes, Firmicutes, Actinobacteria, Deinococcus‐Thermus and Verrucomicrobia (Abdallah et al., 2016; de la Haba, 2011; Sirisena, Ramirez, Steele, & Glamoclija, 2018). Typical Archaea found in salt lakes belong to phylum Euryarchaeota (mainly representatives from Halobacteria class) in addition to some methanogens (Abdallah et al., 2016; Oren et al., 2009; Zhang et al., 2013). It was shown that as salinity increases, Archaea tend to dominate over Bacteria (Simachew et al., 2016).

Despite this progress, little information is available concerning the taxonomic distribution and ecological role of microbes in the Aral Sea. The Aral Sea is an endorheic hypersaline lake in western Central Asia located at the border between Uzbekistan and Kazakhstan. In the mid‐20th century, the lake was one of the largest saline water bodies with 66,000 km^2^ surface area, a total volume of about 1,070 km^3^ and a maximum depth of 66 m. From the 1960s, water has been diverted from rivers leading into the Aral Sea for irrigation of agricultural lands, causing a reduction of the natural flow of water into the Aral Sea by 90%. As a result, the size of the Aral Sea declined and was divided into three separate water bodies (Large Aral Sea, Lake Tshchebas and Small Aral Sea), each with different physicochemical and biological features (Izhitskiy et al., 2016). Desiccation of the Aral Sea continued intensively. The average depth was reduced from 66 m to 16 m and salinity increased from 10 g/L to 120–130 g/L over 54 years (Gaybullaev, Chen, & Kuo, 2012; Rafikov & Mamadjanova, 2014).

There are many reports on the salinity level, temperature fluctuations and physicochemical properties of the Aral Sea water basin (Gaybullaev et al., 2012; Izhitskiy et al., 2016; Rafikov & Mamadjanova, 2014). Although culture‐dependent studies have been reported (Aripov at al., 2016), a comprehensive understanding of the microbial community composition and structure in the Aral Sea remains elusive. The aim of this work was to describe the prokaryotic community of the hypersaline Large Aral Sea (Uzbekistan) assessed by a culture‐independent approach using 16S rRNA gene library analysis. This is the first prokaryotic diversity analysis of a changing hypersaline lake caused by human activities.

## 2 MATERIALS AND METHODS

### 2.1 Study site and sampling

**Figure 1:**
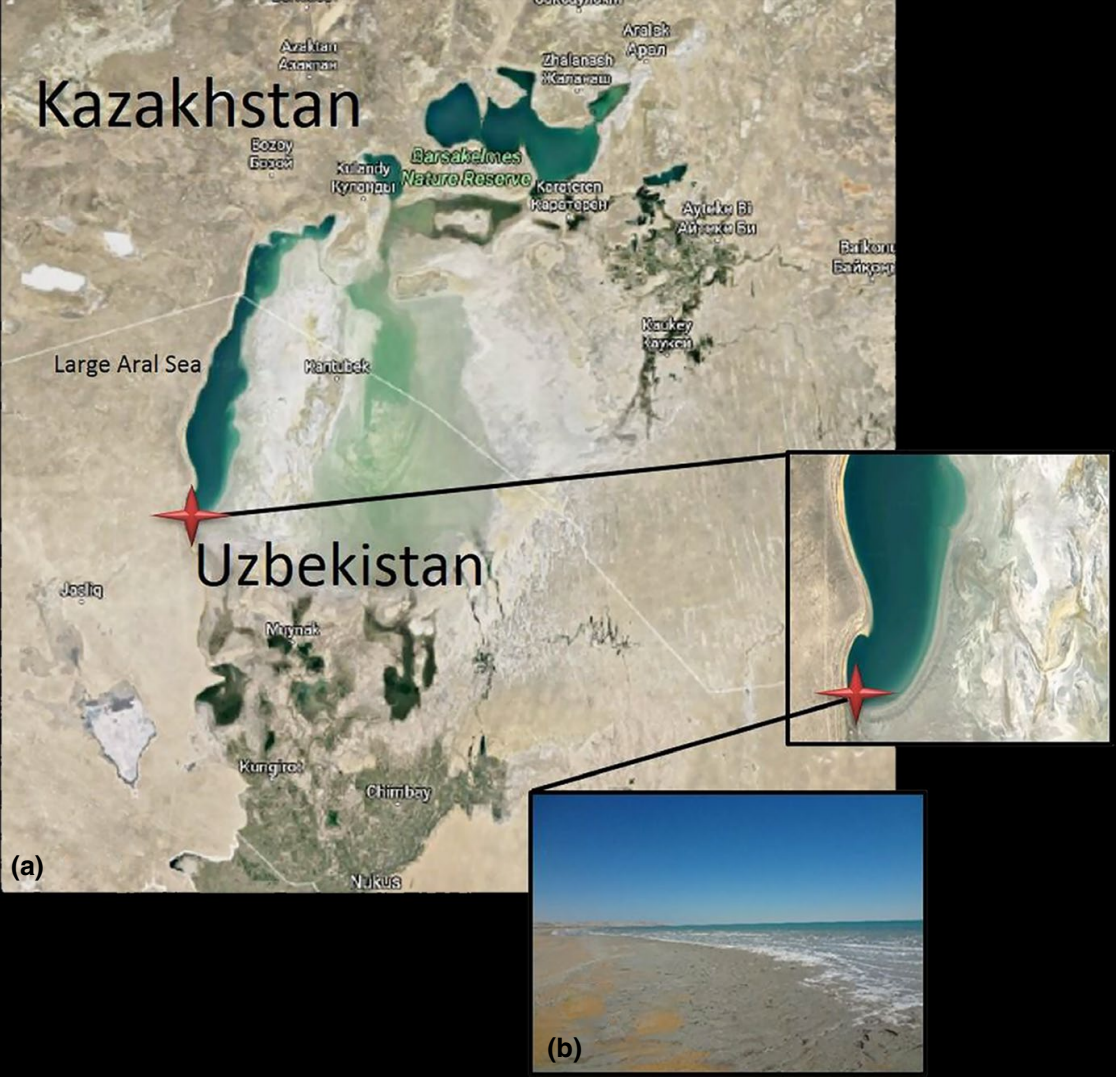
Location of study site. (a) Map of the Aral Sea in 2014 showing location of sampling site in the Large Aral Sea with red mark. (b) Close‐up photograph of the Large Aral Sea beach where sampling was done (44°25′41.5″N, 58°14′34.7″E). Source of map: https://www.google.com/maps

Water samples from the south‐west coastal shallow part (at 25–30 cm depth) of the Large Aral Sea (44°25′41.5″N, 58°14′34.7″E) were collected in July 2014. A map of the Aral Sea and location of the sampling site is shown in Figure 1. The meteorological and weather conditions were stable throughout the survey. Sunny weather with daytime air temperature about 42°C persisted throughout the observation period. Water samples were taken aseptically and transferred to sterile plastic containers and were brought to the laboratory within 1–2 days. Cells were harvested from ~5 L of sample which was prefiltered through paper filters (20 μm) and subsequently filtered through 0.2 μm (Whatman, Germany) nitrocellulose filters until they got clogged. Subsequently, filters with biomass were fixed in RNAlater (Sigma‐Aldrich), and stored at 4°C until analysis in the lab. Filtered surface (0–5 cm) water was used for analysis of chemical composition by a Thermo Scientific^™^ iCAP^™^ 7400 ICP‐OES Duo analyzer. Analyses of major and minor elements in the water revealed the following major element composition (in ppm): Na, 29755; Mg, 9575; S, 5337; K, 1799; Ca, 796; Si, 434; Sr, 74 and the following minor element composition (in ppb): Pb, 164; P, 148; Ti, 17.7; Ba 5.9; Zn 3.9; Mn, 2.6; Fe, 0.1. Salinity and pH was determined, respectively, to 14.0% with a hand refractometer (S/Mill, Atago, Japan) and to 7.7 using a PHM210 pH meter.

### 2.2 DNA isolation

DNA was extracted directly from the filters carrying cells lysed in TE buffer by phenol extraction followed by ethanol precipitation according to a modified cetyltrimethylammoniumbromide (CTAB) protocol (Dempster, Pryor, Francis, Young, & Rogers, 1999). Biomass was suspended in 560 μL of TE buffer (10 mM Tris–HCl, pH 8, 1 mM EDTA). Then 30 μl of 10% SDS, 20 μl of proteinase K (20 mg/ml) and 6 μl of RNAase (10 mg/ml) were added followed by incubation at 55°C for 1 hr. Subsequently, 100 μl of 5 M NaCl and 80 μl of CTAB extraction buffer (10% CTAB in 0.7 M NaCl) were added and the mixture was incubated at 65°C for 20 min. In order to extract DNA from the suspension, an equal volume of chloroform:isoamyl alcohol (24:1 v/v) was added. After centrifugation at 12,000 rpm for 10 min at 20°C, 0.6 ml of cold isopropanol was added to the aqueous phase and gently mixed by hand. The tube was then left at −20°C for 30 min and centrifuged at 12,000 rpm for 15 min at 4°C. The precipitated DNA was washed with 1 ml of 70% ethanol, dried at 37°C for 30 min, and then resuspended in 30 μl of TE buffer and kept overnight at 4°C to dissolve precipitated DNA.

### 2.3 PCR amplification

Extracted DNA was used as template for amplification of 16S rRNA genes by PCR. PCR was performed using a 1000‐Series Thermal Cycler PCR system (BIO‐RAD) with a primary heating step for 2 min at 95°C, followed by 30 cycles of denaturation for 30 s at 95°C, annealing for 30 s at 55°C, and extension from 45 to 90 s (depending on the length of primer sets) at 68°C, then followed by a final extension step for 10 min at 68°C. Each 50 μl reaction mixture contained 10–100 ng of DNA, 10 μl of 5 × PCR buffer, 10 mM of each of the dNTPs (dATP, dGTP, dCTP, and dTTP), 0.5 μl of each primer (25 pmol/ml), 0.2 μl of *Taq* DNA polymerase (1.0 U; Invitrogen), 0.1% bovine serum albumin, and sterile water to a final volume of 50 μl. Universal bacterial and archaeal 16S rRNA gene oligonucleotide primer sets were used (Table 1). PCR amplified products were examined by electrophoresis using a 1% agarose gel containing GelRed (0.5 μg/ml).

**Table 1:** Oligonucleotide primers used for PCR

Target	Position^a^	Oligonucleotide primers sequences (5′‐3′)	References
Bacterial 16S rRNA gene	27F	GAGTTTGATCCTGGCTCA	Rainey, Dorsch, Morgan, and Stackebrandt, (1992)
	1525R	GAAAGGAGGAGATCCAGCC	
Archaeal 16S rRNA gene	21F	TTCCGGTTGATCCYGCCGGA	DeLong (1992)
	958R	YCCGGCGTTGAMTCCAATT	

a Corresponding to 16S rRNA gene sequence position in *Escherichia coli.*

### 2.4 Clone library construction

The obtained PCR products were purified with GenElute^™^ PCR Clean‐up Kit (Sigma) and cloned with TOPO TA cloning kit version O, using chemical transformation according to the manufacturer's instructions (Invitrogen). Two (one for Archaea and the other for Bacteria) 16S rRNA gene libraries were generated. Plasmid DNA from selected clones was purified using GenElute™ Plasmid Mini‐Prep Kit (Sigma) according to the manufacturer's recommendations.

### 2.5 Sequence analysis and bioinformatics

Sanger sequencing of cloned products and purified PCR products was performed using ABI PRISM BigDye 3.1 Terminator Cycle Sequencing Ready Reaction Kit (Applied Biosystems) as described in the manufacturer's protocol. The sequences obtained were edited manually in ChromasLite software Version 2.1.1 for Windows 7 and higher, and merged using EMBOSS Explorer. Chimeric 16S rRNA sequences were detected with DECIPHER and were discarded. 16S rRNA gene sequences were initially compared with reference sequences at NCBI using BLAST (Altschul et al., 1997). Alignment for phylogenetic analysis of 16S rRNA gene sequences was made by using Clustal X2 (Larkin et al., 2007). A phylogenetic tree was constructed using the neighbor joining method with MEGA 6 software (Saitou & Nei, 1987; Tamura, Stecher, Peterson, Filipski, & Kumar, 2013). Bootstrapping analysis using 500 replicates was performed to estimate the confidence of tree topologies (Felsenstein, 1985).

## 3 RESULTS

In order to obtain an overview of the microbial community, one bacterial and one archaeal 16S rRNA gene library were constructed. Twenty‐five clones from each library were analyzed. Most of the obtained clones were phylogenetically associated with environmental clones obtained from similar hypersaline habitats (Tables 2 and 3). As a large fraction of the sequences shared less than 97% similarity to their closest cultivated relatives, the Aral Sea thus harbors a unique microbial community with many novel genera or species. Out of 25 bacterial sequences, 18 OTUs were identified.

**Table 2:** Blast results of closest relatives of bacterial 16S rRNA gene clone sequences obtained from the Large Aral Sea water samples

Phylogenetic Affiliation	Clone sequence, accession number	Closest sequence match, accession number	Original Source of the closest sequence match	Closest species, accession number	% Similarity of, Closest sequence match (species match)
Alphaproteobacteria	B4 MG388257	Uncultured bacterial clone DSFBPENV12bac_5A2, KC465657	Salton Sea geothermal system	*Roseovarius atlanticus*, NR_148630	96.9 (94.7)
	B6 MG388259	Uncultured bacterial clone M6m1‐51 JN092157	Gut microbiome of *Nephrops norvegicus*	*Marivita litorea,* NR_044513	95.5
	B7 MG388260	*“Roseovarius algicolus”,* NR_148335	*Cochlidinium* *polykrikoides* culture fluid	*Roseovarius algicolus,* NR_148335	96.8
	B13 MG388266	Uncultured Rhodobacteraceae EG8*,* AM691101	Canadian hypersaline spring system	*Roseovarius tolerans,* NR_026405	99.8 (98.9)
	B15 MG388268	*Roseibacterium elongatum,* NR_121734	West coast of Australia	*R. elongatum,* NR_121734	96.0
	B16 MG388269	Uncultured Rhodobacter clone LA1‐B32N, AF513928	Lake in The Hawaiian Archipelago,	*Roseovarius pacificus,* KC593284	93.7 (93.1)
	B24 MG388277	*Sulfitobacter delicatus,* NR_025692	Starfish (*Stellaster equestris*)	*Sulfitobacter delicatus,* NR_025692	95.8
Betaproteobacteria	B18 MG388271	Uncultured Achromobacter sp. clone 2SN, EU887771	Digester of Nisargruna biogas plant, India	*Achromobacter denitrificans,* NR_042021	98.7 (96.0)
	B25 MG388278	Uncultured bacterial clone SINI470, HM126867	High mountain lake in the Tibetan Plateau	*Achromobacter pulmonis,* NR_117644	99.4 (96.2)
Gammaproteobacteria	B1 MG388254	Uncultured bacterial clone 7658, KJ546098	Hypersaline ponds of a marine saltern in Santa Pola, Spain	*Spiribacter salinus,* NR_103952	100 (98.1)
	B5 MG388258	Uncultured bacterial clone JS2_B08, KT318689	Northeastern Gulf of Mexico	*Pseudoalteromonas phenolica,* CP013187	96.1
	B9 MG388262	*Idiomarina* sp. TBZ1 EU305725	Hypersaline Urmia Lake in Iran	*Idiomarina* sp. TBZ1, EU305725	100
	B12 MG388265	Uncultured gamma proteobacterial clone HAHS13, HQ397064	Haloalkaline soil, India	*Bradymonas sediminis,* KM034744	88.5 (85.8)
	B14 MG388267	Uncultured bacterial clone 7658, KJ546098	Hypersaline ponds of a marine saltern in Santa Pola, Spain	*Spiribacter curvatus,* NR_145955	100 (97.7)
	B20 MG388273	Uncultured bacterial clone 7658, KJ546098	Hypersaline ponds of a marine saltern in Santa Pola, Spain	*S. curvatus,* NR_145955	99.9 (97.5)
	B21 MG388274	*Idiomarina* sp. TBZ1, EU305725	Hypersaline Urmia Lake in Iran	*Idiomarina* sp. TBZ1, EU305725	98.7
Actinobacteria	B8 MG388261	*Pontimonas salivibrio* NR_109611	seawater reservoir of a solar saltern in Korea	*P. salivibrio,* NR_109611	96.2
	B17 MG388270	Uncultured bacterial clone SINI711, HM127059	high mountain lake in the Tibetan Plateau	“*Candidatus* Planctoluna difficilis”*,* NR_125495.1	99.1 (96.0)
	B19 MG388272	Uncultured bacterial clone SINI711, HM127059	high mountain lake in the Tibetan Plateau	“*Candidatus* Planctoluna difficilis”*,* NR_125495.1	97.5 (94.5)
	B22 MG388275	Uncultured bacterial clone SINI1037, HM126703	high mountain lake in the Tibetan Plateau	*P. salivibrio,* NR_109611	90.5
	B23 MG388276	Uncultured bacterial clone SINI711, HM127059	high mountain lake in the Tibetan Plateau	“*Candidatus* Planctoluna difficilis”*,* NR_125495.1	99.7 (96.2)
Bacteroidetes	B2 MG388255	Uncultured bacterial clone SINH641, HM128159	high mountain lake in the Tibetan Plateau	*Phaeodactylibacter luteus,* NR_136808	100 (85.4)
	B3 MG388256	*Haliscomenobacter hydrossis,* NR_074420	high mountain lake in the Tibetan Plateau	*H. hydrossis,* NR_074420	96.4
	B10 MG388263	Uncultured bacterial clone SINH641, HM128159	high mountain lake in the Tibetan Plateau	*P. luteus,* NR_136808	99.8 (83.9)
	B11 MG388264	Uncultured bacterial clone E6aH07, DQ103641	hypersaline endoevaporitic microbial mat, USA	*“Flavobacterium kamogawaensis”,* AB275998	94.2 (87.0)

**Table 3:** Blast results of closest relatives of archaeal 16S rRNA gene clone sequences obtained from the Large Aral Sea water samples

Clone Sequence, accession number	Closest sequence match, accession number	Original Source of the closest Sequence match	Closest species, accession number	% Similarity, of closest sequence match (species match)
A1 MG388228	Uncultured archaeon clone MHNAA10, HQ157569	Sfax coastal solar salterns, Tunisia	*Halogeometricum rufum* NR_113450	99.8 (92.6)
A8 MG388235	Uncultured archaeon clone MHNAA25, HQ157584	Sfax coastal solar salterns, Tunisia	*H. rufum* NR_113450	98.7 (91.7)
A9 MG388236	Uncultured archaeon clone MHNAA10, HQ157569	Sfax coastal solar salterns, Tunisia	*H. rufum* NR_113450	99.1 (91.8)
A22 MG388249	Uncultured archaeon clone MHNAA8, HQ157587	Sfax coastal solar salterns, Tunisia	*H. rufum* NR_113450	99.3 (90.2)
A20 MG388247	Uncultured archaeon clone 186ZD11, CU467225	Tunisian multipond solar saltern	*H. rufum* NR_113450	99.0 (91.6)
A3 MG388230	Uncultured archaeon clone 186ZD11, CU467225	Tunisian multipond solar saltern	*H. rufum* NR_113450	99.8 (92.4)
A4 MG388231	Uncultured archaeon clone 186ZD11, CU467225	Tunisian multipond solar saltern	*H. rufum* NR_113450	99.8 (92.4)
A7 MG388234	Uncultured archaeon clone 186ZD08, CU467228	Tunisian multipond solar saltern	*H. rufum* NR_113450	99.8 (92.6)
A11 MG388238	Uncultured archaeon clone 186ZD11, CU467225	Tunisian multipond solar saltern	*H. rufum* NR_113450	99.3 (91.9)
A2 MG388229	Uncultured archaeon clone 2009, KJ546110	Aquatic hypersaline environments, Spain	*H. rufum* NR_113450	99.7 (92.9)
A13 MG388240	Uncultured archaeon clone 2009, KJ546110	Aquatic hypersaline environments, Spain	*H. rufum* NR_113450	99.7 (92.6)
A15 MG388242	Uncultured archaeon clone 2009, KJ546110	Aquatic hypersaline environments, Spain	*H. rufum* NR_113450	99.1 (91.8)
A16 MG388243	Uncultured archaeon clone 2009, KJ546110	Aquatic hypersaline environments, Spain	*H. rufum* NR_113450	99.4 (92.1)
A18 MG388245	Uncultured archaeon clone 2009, KJ546110	Aquatic hypersaline environments, Spain	*H. rufum* NR_113450	99.4 (92.2)
A21 MG388248	Uncultured archaeon clone 2009, KJ546110	Aquatic hypersaline environments, Spain	*H. rufum* NR_113450	99.1 (91.8)
A23 MG388250	Uncultured archaeon clone 2009, KJ546110	Aquatic hypersaline environments, Spain	*H. rufum* NR_113450	99.2 (91.6)
A24 MG388251	Uncultured archaeon clone 2009, KJ546110	Aquatic hypersaline environments, Spain	*H. rufum* NR_113450	99.8 (92.2)
A5 MG388232	Uncultured archaeon clone MHNAA19, HQ157578	Sfax coastal solar salterns, Tunisia	*Halogeometricum limi* NR_113493	99.5 (92.0)
A10 MG388237	Uncultured archaeon clone MLNAA12, HQ157592	Sfax coastal solar salterns, Tunisia	*H. limi* NR_113493	99.2 (92.3)
A14 MG388241	Uncultured archaeon clone 186ZC08, CU467239	Tunisian multipond solar saltern	*H. limi* NR_113493	99.4 (92.0)
A17 MG388244	Uncultured archaeon clone 186ZD01, CU467234	Tunisian multipond solar saltern	*H. limi* NR_113493	98.4 (91.8)
A6 MG388233	Uncultured archaeon clone 2009, KJ546110	Aquatic hypersaline environments, Spain	*Halobellus inordinatus* AB935405	99.7 (92.6)
A12 MG388239	Uncultured archaeon clone 06245, KJ546103	Hypersaline ponds of a marine saltern, Spain	*H. inordinatus* AB935405	99.6 (92.2)
A19 MG388246	Uncultured archaeon clone MHNAA8, HQ157587	Sfax coastal solar salterns, Tunisia	*H. inordinatus* AB935405	97.4 (90.4)
A25 MG388252	Uncultured archaeon clone 2009, KJ546110	Aquatic hypersaline environments, Spain	*H. inordinatus* AB935405	99.3 (90.0)

**Figure 2:**
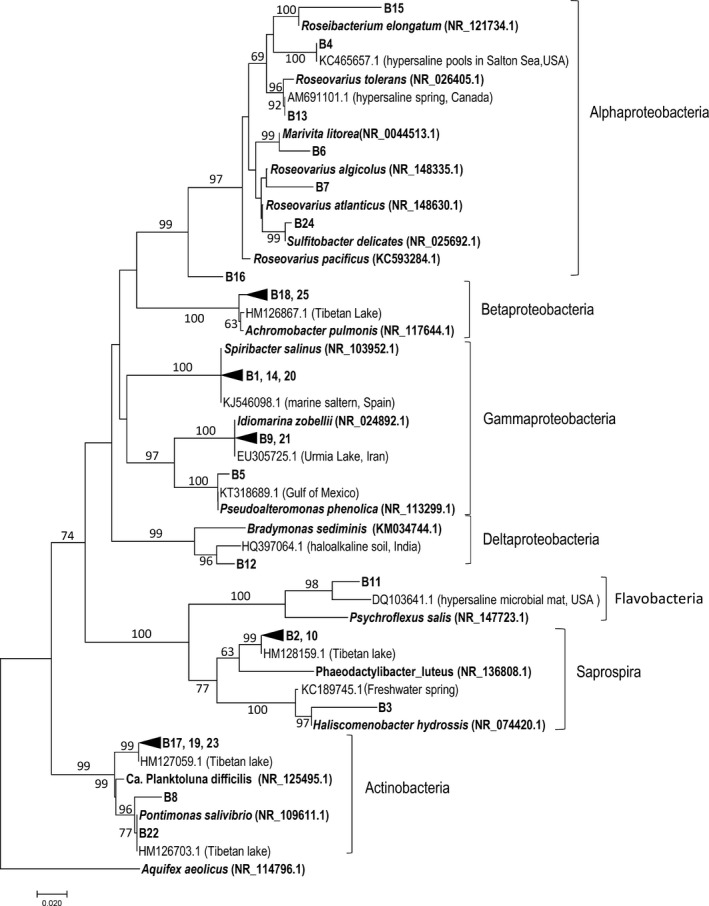
Neighbor‐Joining phylogenetic tree showing the phylogenetic positions of the bacterial 16S rRNA clone sequences (B1–B25). The closest cultivated bacteria are indicated in bold. The Aral Sea sequences comprising OTUs are collapsed. Closest related environmental sequences are also included. Database accession numbers are given in brackets. Bootstrap values ≥63% are indicated at branch nodes and based on 500 iterations. Positions containing gaps or missing data were excluded from the analysis. The tree was rooted using the deep‐branching *Aquifex aeolicus* as outgroup. The bar indicates the number of base substitutions per site

Taxonomic analysis revealed a predominance of Proteobacterial phyla (64% of the clones) followed by Actinobacteria and Bacteroidetes. Within Proteobacteria, representatives from Alphaproteobacteria, Betaproteobacteria and Gammaproteobacteria were found. The alphaproteobacterial sequences all clustered among the “Rhodobacteraceae” (Figure 2), with phylogenetic connections to genera *Roseibacterium*, *Roseovarius, Marivita* and *Sulfitobacter*. Representatives of these genera have been isolated from marine and hypersaline habitats worldwide, including *Roseovarius tolerans* recovered from a hypersaline, heliothermal meromictic lake in Antarctica (Labrenz et al., 1999), sharing 98.9% identity with clone B13. Some of the clones also share similarities with bacteria isolated from lobster and starfish (B6 and B15).

Only two clones (B6 and B15) belonged to Betaproteobacteria. They clustered within the *Achromobacter* genus (order *Burkholderiales*) and were highly related to environmental sequences from a Tibetan lake. *Achromobacter* spp. have not been recovered from marine or hypersaline habitats, but mostly from human and soil. The Gammaproteobacteria clones were affiliated with the genera *Idiomarina, Pseudoalteromonas* and *Spiribacter*. *Spiribacter* spp. are common in marine salterns and similar habitats (Fernandez et al., 2013; Leon et al., 2013, 2015). Two Gammaproteobacteria clones (B9 and B21) showed very close match to *Idiomarina* spp., including an isolate from the hypersaline Urmia Lake in Iran (Vahed et al., 2011). Clone B‐12 was particularly divergent, as it shared only 85.5% identity with its closest cultured relative, *Bradymonas sediminis,* a marine deltaproteobacterium isolated from a coastal sediment in China and belonging to a recently described order, Bradymonadales (Wang, Liu, Zhao, Du, & Chen, 2015). Slightly higher similarity (88.5% ID) was found to an environmental sequence from haloalkaline soil in India, suggesting that this bacterium might be widespread.

The Actinobacteria clones were most related (90.5%–99.7%) to environmental sequences from a Tibetan lake (Zhang et al., 2013). The closest cultured relative was *Pontimonas salivibrio* isolated from the seawater reservoir of a solar saltern in Korea (Jang, Cho, & Cho, 2013) and the freshwater genus “*Candidatus* Planktoluna” (Hahn, 2009). The Bacteroidetes clones affiliated with phyla Saprospira and Flavobacteriia, two of which were closely related to clone sequences from a Tibetan lake (Zhang et al., 2013). The rest of the Bacteroidetes clones matched to *Haliscomenobacter hydrossis*, isolated from activated sludge (van Veen, Kooij, Geuze, & Vlies, 1973) and *Psychroflexus salis*, a halophile from a salt lake in China (Zhong et al., 2016).

**Figure 3:**
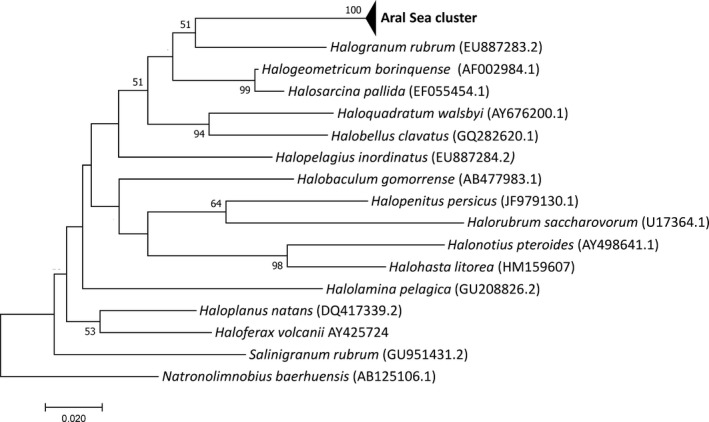
Neighbor‐Joining phylogenetic tree showing the position of the archaeal 16S rRNA clone sequences (the Aral Sea cluster) within the Haloferacaceae family as defined by Gupta et al. (2015). Only type strains of type species are included as references, with accession numbers shown in brackets. Bootstrap values ≥51% are indicated at branch nodes and based on 500 iterations. Positions containing gaps or missing data were excluded from the analysis. *Natronolimnobius baerhuensis*, belonging to the closest related family, Natrialbaceae*,* was used as outgroup. The bar indicates the number of base substitutions per site

The archaeal sequences were all related and formed a tight cluster within the Haloferacaceae family in the class Halobacteria, sharing sequence similarity with the genera *Halogeometricum* and *Halobellus* (Table 3, Figure 3). The sequence identity with closest cultivated species ranged, however, only from 90.0% to 92.9%, demonstrating a striking uniqueness of the archaeal community with only novel species. The sequence similarities with environmental clone libraries were, however, much higher (97.4–99.8% ID), and matched particularly with sequences obtained from solar salterns and other aquatic hypersaline habitats in Spain and Tunisia.

## 4 DISCUSSION

Chemical analyses of water samples showed that the Large Aral Sea is comparable to natural hypersaline lake ecosystems in terms of salinity and mineral composition (Izhitskiy et al., 2016). In the present study, the microbial diversity of the Large Aral Sea was investigated for the first time by culture‐independent methods. 16S rRNA clone library analysis was performed to assess the bacterial and archaeal community structures.

The results from the analysis of bacterial clone library indicated a large diversity with identification of members from several additional classes (Figure 2, Table 2), all belonging to Proteobacteria, Bacteroidetes or Actinobacteria. The proteobacterial sequences were related to environmental sequences previously reported in different saline lakes, salterns and other hypersaline ecosystems worldwide, where these bacteria participate in biogeochemical cycles of biogenic elements under aerobic and anaerobic conditions (Edwardson & Hollibaugh, 2018; Paul et al., 2016; Sirisena et al., 2018). A high species richness of Alphaproteobacteria and Gammaproteobacteria observed in the Aral Sea is consistent with findings from similar environments, such as the hypersaline Urmia Lake in Iran (Vahed et al., 2011), as well as with findings from ocean and marine waters, solar salterns (Fernandez et al., 2013) and other high‐salinity environments. *Roseovarius* and *Spiribacter* were the most abundant genera within Alphaproteobacteria and Gammaproteobacteria, respectively. Previous studies have also reported representatives of *Spiribacter* to be one of the most abundant bacterial genera in various salterns (Edwardson & Hollibaugh, 2018; Fernandez et al., 2013). Next to *Spiribacter*, representatives of the genus *Idiomarina* were abundant in Aral Sea water. Presence of *Idiomarina* spp. was also reported from Urmia Lake (Vahed et al., 2011). Within Betaproteobacteria, representatives of the genus *Achromobacter* were identified. Members of this phylum have been found in hypersaline environments of different characteristics around the world including a high mountain salt lake in the Tibetan Plateau and other hypersaline environments (Mutlu et al., 2008). In accordance with studies of similar habitats, our results also indicated an abundance of Actinobacteria and Bacteroidetes, with representatives of genera *Rhodoluna* and *Pontimonas,* and *Phaeodactylibacter*, respectively. Surprisingly, most of the Actinobacterial and Bacteroidetes sequences shared highest similarity with environmental sequences from high mountain salt lakes in the Tibetan Plateau (Zhang et al., 2013). The absence of cyanobacterial sequences is striking and indicates domination of heterotrophic life‐style in the Aral Sea.

The Archaeal 16S rRNA clone library indicated that species of the genus *Halogeometricum* are abundant components of the prokaryotic community. This supports the view that *Halogeometricum* is widespread and often dominates within the microbial community in hypersaline environments (Baati et al., 2008; Ghai et al., 2011; Trigui et al., 2011). Next to *Halogeometricum*, representatives of the genus *Halobellus* were the dominant group of Archaea. These observations confirm that representatives of the genera *Halogeometricum* and *Halobellus* are well adapted to extremely high salt concentrations.

Being a hypersaline environment, the Aral Sea has a diverse halotolerant and halophilic microbial community. While it is not possible to predict their metabolism from 16S rRNA sequences alone, the closest phylogenetic affiliations were found mostly to aerobic and anaerobic heterotrophs. As a dominating group, Proteobacteria are known for their ubiquity and metabolic flexibility which includes their ability to tolerate extreme and/or oligotrophic environments, utilize diverse carbon compounds and to maintain aerobic and anaerobic lifestyles. Sulfur‐metabolizing bacteria such as members of*Sulfitobacter* were also detected as minor populations and could utilize various inorganic sulfur compounds as electron donors. Bacterial members among *Achromobacter* genus are particularly remarkable in their ability to grow in anaerobic as well as aerobic conditions and are well‐known nitrate‐reducing organisms. All these microbes are important players for cycling of carbon, nitrogen and sulfur.

The Aral Sea appears to harbor unique microbial populations as a large fraction of 16S rRNA sequences shared less than 97% identity with their closest cultivated relatives and thus likely represent novel species or genera (Tables 2 and 3). This environment can therefore be regarded as a rich source of novel prokaryotic taxa. Only one of the sequences from the clone libraries (B9) was identical (across 1,381 bases) to a previously cultivated organism, an *Idiomarina* sp. isolate from the hypersaline Urmia Lake in Iran (Vahed et al., 2011). Members of this genus are mostly found in seawater, but have also been recovered from hypersaline environments (Choi & Cho, 2005; Kwon et al., 2006; Lee, Kim, Yun, & Whang, 2015; Martinez‐Canovas, Bejar, Martinez‐Checa, Paez, & Quesada, 2004; Vahed et al., 2011; Yoon, Jung, Jung, & Oh, 2007; Zhong et al., 2014). The sequence identity of clone B6 with a bacterium from another hypersaline lake in Central Asia suggests a local clonal distribution and a possible biogeographic population structure. Although the long‐standing Baas‐Becking postulate, “Everything is everywhere, but the environment selects” (Baas‐Becking, 1934), has been a cornerstone hypothesis for the evolution and diversification of prokaryotes for decades and is supported by a number of studies (Brewer, Handley, Carini, Gilbert, & Fierer, 2016; Glöckner et al., 2000; Massana, DeLong, & Pedrós‐Alió, 2000); patterns of biogeographic structure among prokaryotes have recently been reported. This has in particular been shown for members of thermo‐acidophilic Archaea inhabiting terrestrial acidic hot springs (Reno, Held, Fields, Burke, & Whitaker, 2009; Whitaker, Grogan, & Taylor, 2003), believed to represent isolated islands enabling evolution through an isolation‐by‐distance mechanism. A similar scenario might apply to hypersaline lakes, although these environments are much larger, with lower geographic barriers, and exchange and dispersal of microbes is thus more easily envisaged, for example, through migrating birds or aerial transportation of dust particles. Dispersal of microbes between Lake Urmia and The Aral Sea, approximately 1,300 km apart is thus plausible. Another interesting issue regarding biogeographic pattern is the abundance of Actinobacteria and Bacteroides sequences from the Aral Sea related to environmental clones from Tibetan high mountain lakes. Two of the Bacteroidetes clones (B2 and B10) share 99.8%–100% identity (maximum one base mismatch) with a number of clones from the Xiaochadan and Chaerhan lakes, located at altitudes of 2,678 and 3,171 m in the Tibetan Plateau, and with salinities of at least 160 and 280 g/L, respectively (Zhang et al., 2013). The long distance and other geographical barriers that is, mountain ranges make direct dispersal of microorganisms from the Plateau of Tibet to the Aral Sea less likely, and may indicate that these organisms represent a Central Asian ecotype with a non‐random distribution.

## 5 CONCLUSION

The molecular approach applied in this study has several potential biases. Most importantly, the data set is not large enough for a thorough community analysis, but does provide a reliable first estimate of the major taxa of the microbial community. The colonization of the Aral Sea by extremophiles during its gradual conversion from a freshwater to a hypersaline lake over a period of 50 years is an interesting issue, with a great potential for providing clues to regional and global dissemination of microbes and factors controlling biogeographic structuring of prokaryotes. The current study represents a “snapshot” in time and space, and the microbial diversity may change with seasons and continuing desiccation or rainfall. However, it provides valuable information regarding the diversity of extremophiles that have colonized the Aral Sea and it is the very first report of the microbial diversity in this man‐made extreme environment.

## CONFLICT OF INTERESTS

None declared.

## AUTHOR CONTRIBUTIONS

NKB, HP, DE and KD conceived and designed the experiments. NKB, HP and DE did the fieldwork. VS and AH performed the laboratory work. VS, AH, NKB, HP and DE analyzed the results. All authors assisted in writing the manuscript.

## ETHICS STATEMENT

None required.

## Data Availability

The authors declare that the experimental data published in this paper are made accessible upon request for interested readers. All 16S rRNA gene sequences of the new strains can be found under accession numbers MG388228‐MG388252 and MG388254‐MG388278 in GenBank.
