# Supplementary material for: A glimpse of the prokaryotic diversity of the Large Aral Sea reveals novel extremophilic bacterial and archaeal groups
Source: Microbiologyopen. 2019 May 6;8(9):e00850. doi: 10.1002/mbo3.850 (PMC6741134; doi:10.1002/mbo3.850)
Supplement: Supplementary file 1 [file MBO3-8-e00850-s001.pdf]

Supplementary materials

A glimpse of the prokaryotic diversity of the Large Aral Sea reveals novel extremophilic bacterial and archaeal groups

Vyacheslav Shurigin[1,2], Anna Hakobyan[1,3†], Hovik Panosyan[3], Dilfuza Egamberdieva[2,4,5], Kakhramon Davranov[2] and Nils-Kåre Birkeland[1*]

[1] Department of Biological Sciences, University of Bergen, P.O. Box 7803, NO-5020 Bergen, Norway;

[2] Department of Microbiology, Faculty of Biology, National University of Uzbekistan, 100174, Tashkent, Uzbekistan;

[3] Department of Biochemistry, Microbiology and Biotechnology, Yerevan State University, 0025, Yerevan, Armenia;

[4] Key Laboratory of Biogeography and Bioresource in Arid Land, Xinjiang Institute of Ecology and Geography, CAS, Urumqi, People's Republic of China

[5] Leibniz Centre for Agricultural Landscape Research (ZALF), 15374 Müncheberg, Germany

[†] Present address: Max Planck Institute for Terrestrial Microbiology, Karl-von-Frisch Str. 10, 35043 Marburg, Germany

Supplementary figure

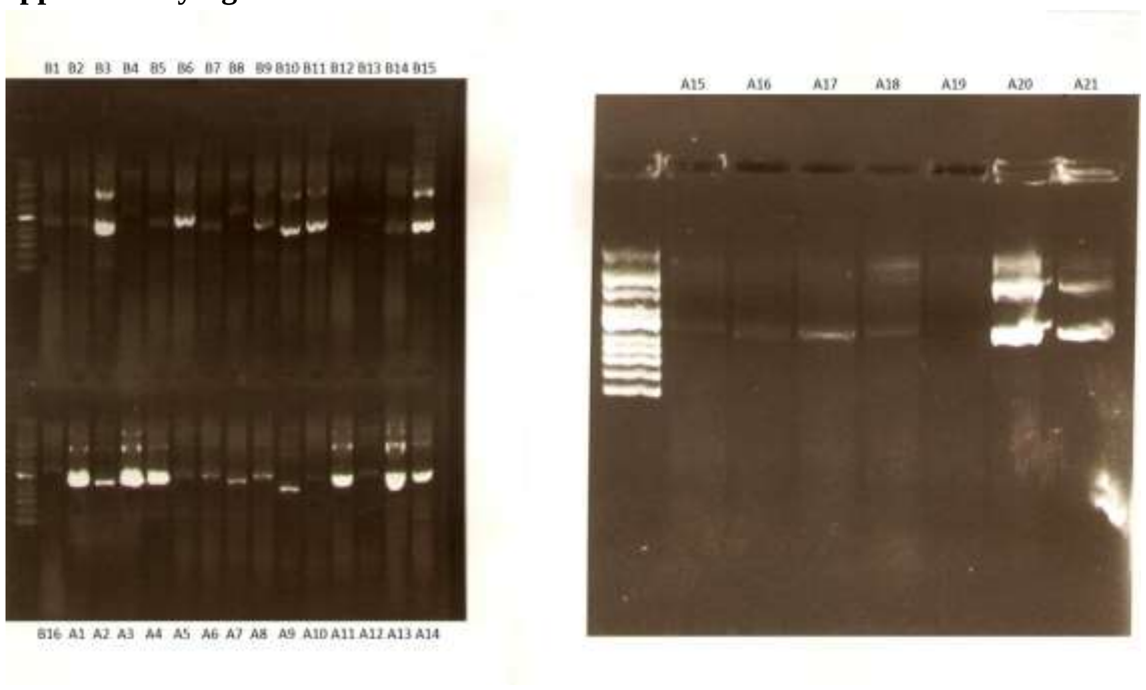

Fig. S1. Agarose gel electrophoretic analysis of 16S rRNA gene PCR amplicons using plasmids from the bacterial (B1-16) and archaeal (A1-21) clone libraries as template DNA. The first lane is 1Kb+ ladder.
